# General Epidemiological Parameters of Viral Hepatitis A, B, C, and E in Six Regions of China: A Cross-Sectional Study in 2007

**DOI:** 10.1371/journal.pone.0008467

**Published:** 2009-12-24

**Authors:** Jian Lu, Yongdong Zhou, Xiaojing Lin, Yongzhen Jiang, Ruiguang Tian, Yonghui Zhang, Jia Wu, Fengwei Zhang, Yong Zhang, Yue Wang, Shengli Bi

**Affiliations:** National Institute for Viral Disease Control and Prevention, Chinese Center for Disease Control and Prevention, Xuanwu District, Beijing, People's Republic of China; Beijing Institute of Infectious Diseases, China

## Abstract

**Background:**

Viral hepatitis is a serious health burden worldwide. To date, few reports have addressed the prevalence of hepatitis A, B, C, and E in China. Therefore, the general epidemiological parameters of viral hepatitis remain unknown.

**Principal Findings:**

In this cross-sectional study, we performed a serological prevalence analysis of viral hepatitis A, B, C, and E in 8,762 randomly selected Chinese subjects, which represented six areas of China. The overall prevalence of anti-Hepatitis C virus antibody (anti-HCV) was 0.58%, which was much lower than was estimated by WHO. The prevalences of Hepatitis B virus surface antigen (HBsAg), anti-Hepatitis B virus surface protein antibody (HBsAb), and anti-Hepatitis B virus core protein antibody (HBcAb) were 5.84%, 41.31%, and 35.92%, respectively, whereas in the group of subjects less than 5 years old, these prevalences were 1.16%, 46.77%, and 8.69% respectively, which suggests that the Hepatitis B virus (HBV)-carrier population is decreasing, and the nationwide HBV vaccine program has contributed to the lowered HBV prevalence in the younger generation in China. Meanwhile, a large deficit remains in coverage provided by the national HBV immune program. In addition, our data suggested the possibility that HBsAb may not last long enough to protect people from HBV infection throughout life. The overall prevalence of anti-Hepatitis A virus antibody (anti-HAV) and anti-Hepatitis E virus antibody (anti-HEV) were as high as 72.87% and 17.66%, respectively. The indices increased with age, which suggests that a large proportion of Chinese adults are protected by latent infection. Furthermore, the pattern of HEV infection was significantly different among ethnic groups in China.

**Conclusions:**

Our study provided much important information concerning hepatitis A, B, C, and E prevalence in China and will contribute to worldwide oversight of viral hepatitis.

## Introduction

Viral infectious diseases are one of the significant challenges to human healthcare. The increased frequency of emerging new infectious viral diseases with high mortality in the last decade, such as SARS, avian influenza A (H5N1), and the most recent “swine flu,” has raised public concern over viral infectious diseases [1]–[4]. Viral hepatitis has threatened human health for a long time with more than one etiologic agent, but this was not recognized until the 1970s. As a class, these agents include mainly HAV, HBV, HCV, Hepatitis D virus (HDV), and HEV; of these, HDV (excluded in this report) only exists with co-infection with HBV [5]–[9]. The four hepatitis etiologic agents belong to entirely different families of viruses. HAV and HEV always cause acute hepatitis that never develops into a chronic disease, whereas HCV and HBV always cause chronic hepatitis and may lead to the development of cirrhosis and hepatocellular carcinoma [5]–[9].

HAV is classified in the family *Picornaviridae* genus *Hepatovirus*, with seven genotypes but one serotype, and is transmitted via the fecal–oral routes [10]–[13]. Hepatitis A outbreaks occur continually, with an estimated 1.4 million new infections worldwide annually [12], and it has decreased in developed regions due to improved personal and public hygiene and efficient vaccination. In developing countries, the disease is related to raw food or contaminated water, which can cause an outbreak at any time [11].

HEV is classified in the family *Hepeviridae* genus *Hepevirus*, with four major genotypes but one serotype. It has a transmission route similar to that for HAV and is prevalent in developing countries [9], [14]–[16]. Fulminant hepatitis E has a mortality rate of 0.5–4.0% in the general population, except in pregnant women, who may suffer up to 20% mortality [14]–[16]. Although a new and effective recombinant vaccine has recently been developed and tested in the US [17], in developing countries, Hepatitis E remains an important health problem.

HBV is classified in the family *Hepadnaviridae* genus *Orthohepadnavirus*, with eight genotypes but one serotype. The virus is transmitted mainly via blood, body-fluid contact, and vertical transmission [18], [19]. Hepatitis B is estimated to infect 2 billion people globally, with 350 million chronic cases and 60,000 deaths each year ^18^. Hepatitis B vaccine has been available since 1982 and has a high efficacy in the prevention of HBV transmission [19], [20]. Because China is particularly hard hit by HBV, the Chinese government has made strong efforts to establish a universal infant immunization program since 1992. Since then, hepatitis B vaccine coverage has reportedly increased from 30.0% for newborns in 1992 to 93.4% in 2005 [21].

HCV is classified in the family *Flaviviridae* genus *hepacivirus*, with six major genotypes and multiple serotypes. It was first identified in 1989 and can be transmitted in a manner similar to HBV [22]–[24]. Hepatitis C is generally asymptomatic, with a strong tendency (up to 80%) for progression to persistent infection [24]–[27]. Both HCV and HBV chronically infected patients may progress to cirrhosis and hepatocellular carcinoma [5]. Unfortunately, no vaccine is available to date [28], [29]. According to estimates from WHO, that over 170 million people have been infected with HCV, 3% of the population of the world, with 3 to 4 million new infections per year and a prevalence rate of 3.9% in the western pacific region since 1998 [22].

In China, viral hepatitis poses a major public health problem. Owing to it's long history, large population, environmental pollution, and poor hygiene, hepatitis A and E outbreaks and sporadic incidents occur regularly [11], [30]. To date, few investigations of the epidemiology of hepatitis A and E have been performed, and no complete and precise studies have been reported. Even with the high rate of hepatitis B, only rare large-scale population-based studies exist [21]. Furthermore, the 13-year-long prevention effort involving the vaccination of children against HBV has not been well assessed. With regard to hepatitis C, given the WHO estimate of 170 million cases of HCV and 350 million of HBV [18], [22], it is reasonable to speculate that HCV cases should be about half as common clinically as HBV cases. However, these results are not consistent with the real clinical situation in China [31]. Furthermore, few large-scale epidemic data exists to assess the true HCV burden in China and worldwide. Simply put, viral hepatitis accounts for a very large proportion of viral diseases in China and the world, notwithstanding newly emerged viruses for which reliable large-scale epidemiological surveys and surveillance statistics are lacking.

In this study, in an effort to examine the above-mentioned issues, we performed a serological cross-sectional investigation of prevalence of hepatitis A, B, C, and E in 8,762 individuals throughout Sichuan, Qinghai, Ningxia, Gansu, and Helongjiang Provinces, as well as in Beijing city. Our study may unravel the true status of viral hepatitis and provide valuable information about global surveillance and estimation.

## Results

### Prevalence of Anti-HCV

**Figure 1:**
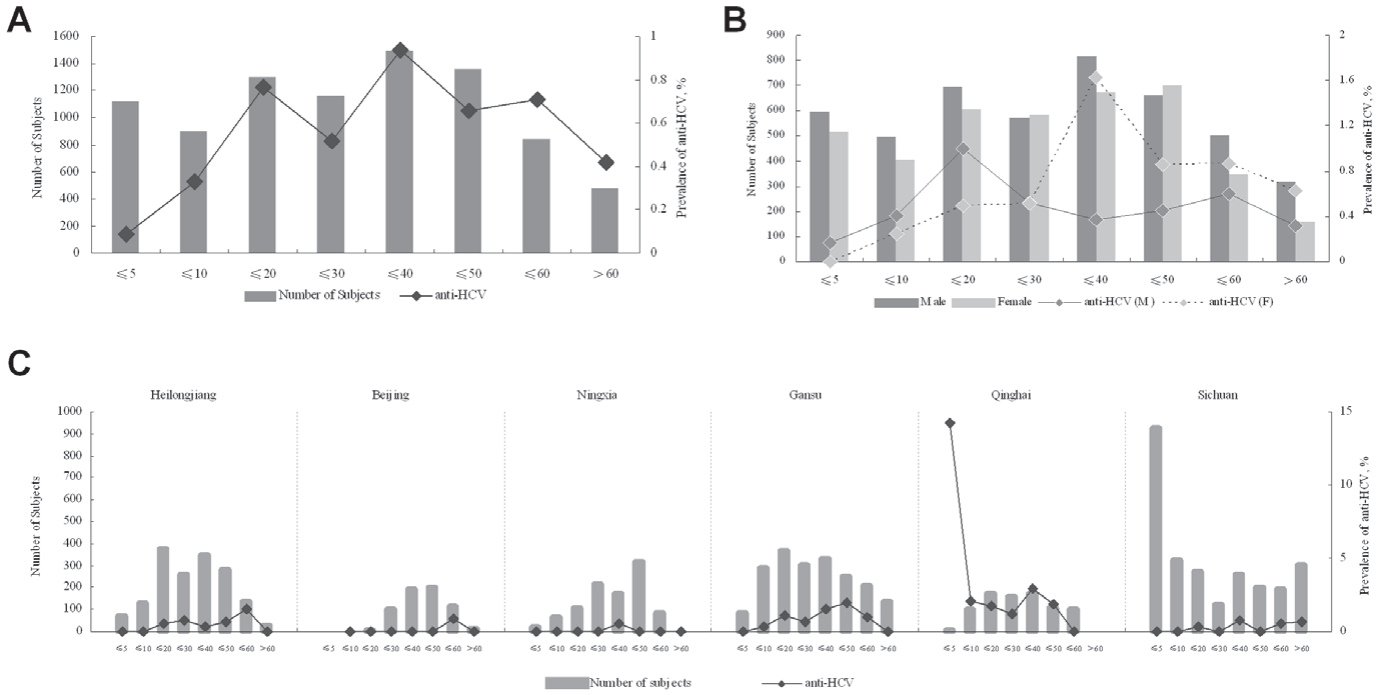
Serological features of anti-HCV prevalence in China. Bars represent the number of subjects in each group indicated by left vertical axis, and the points represent the prevalence in each group indicated by right vertical axis. The horizontal axis represents the age ranges. A, Overall characterization of anti-HCV by age group. B, Overall characterization of anti-HCV by gender, M, male, F, female. C, Regional characterization of anti-HCV by age group.

Overall prevalence of anti-HCV in 8,762 subjects was 0.58% (only 51 positive), and the prevalence of anti-HCV in the ≤5, ≤10, ≤20, ≤30, ≤40, ≤50, ≤60 and >60 age groups was 0.09%, 0.33%, 0.77%, 0.52%, 0.94%, 0.66%, 0.71%, and 0.42%, respectively. For those subjects aged between 10 and 60 years of age, the anti-HCV prevalence was at a relatively high level (Fig. 1A). For age groups younger than 10, the anti-HCV prevalence was much lower than in older groups (only one positive was found in 1,116 subjects in the ≤5 group overall), for two main reasons. First, since HCV identification began in 1989, blood donor screening for HCV has reduced iatrogenic transmission through blood transfusion, transplantation, and nosocomial infections resulting from inadequately sterilized equipments [32]. Second, except for vertical and perinatal transmission, as age increases, the probability of older groups contacting HCV becomes greater than for younger groups (Fig. 1A). However, in the >60 age group, anti-HCV prevalence was also lower, which may be partially explained by many HCV-related cirrhosis and hepatocellular carcinoma deaths in this group in China (Fig. 1A) [33].

The overall prevalence of anti-HCV in 4101 female subjects was 0.68% (28 positive) which was higher than that in the 4661 male subjects, 0.49% (23 positive). For those subjects younger than 30, the prevalence of anti-HCV in female was lower than that in male, while this prevalence increased and turned to be higher than that in male in >30 age groups (Fig. 1B).

With regard to regional differences in the prevalence of anti-HCV, of 1,647 subjects from Heilongjiang Province, the local prevalence was 0.55%, consistent with the overall prevalence described above. The prevalence of anti-HCV in the ≤30 and the ≤60 groups, 0.77% and 1.47%, respectively, was higher than that in the other age groups (Fig. 1C). In Beijing and Ningxia, the regional prevalence was 0.16% of 636 subjects and 0.10% of 998 subjects, respectively (Fig. 1C). In Gansu, the regional prevalence was 0.96% among 1,986 subjects. All 87 subjects in the ≤5 group tested negative for anti-HCV, with all positive cases found among subjects between 5 and 60. The older age range had the highest rates, with a peak of 1.97% in the ≤50 group, and the younger age range showed a small peak of 1.09% in the ≤20 group (Fig. 1C). The prevalence of anti-HCV in Qinghai was significantly higher (1.84%) than in other regions, with the ≤40 group reaching 2.92%. One positive case was found in the ≤5 group, accounting for 14.29% of seven subjects in this age group in this region. The subject was a boy of one year of age who tested positive for HBsAb and HBcAb simultaneously. In Sichuan, 930 subjects in the ≤5 group were tested, and all were negative. Positive results appeared in the ≤20, ≤40, ≤60, >60 groups, with prevalences of 0.36%, 0.76%, 0.51%, and 0.66%, respectively.

### Prevalence of Serologic Markers of HBV

**Figure 2:**
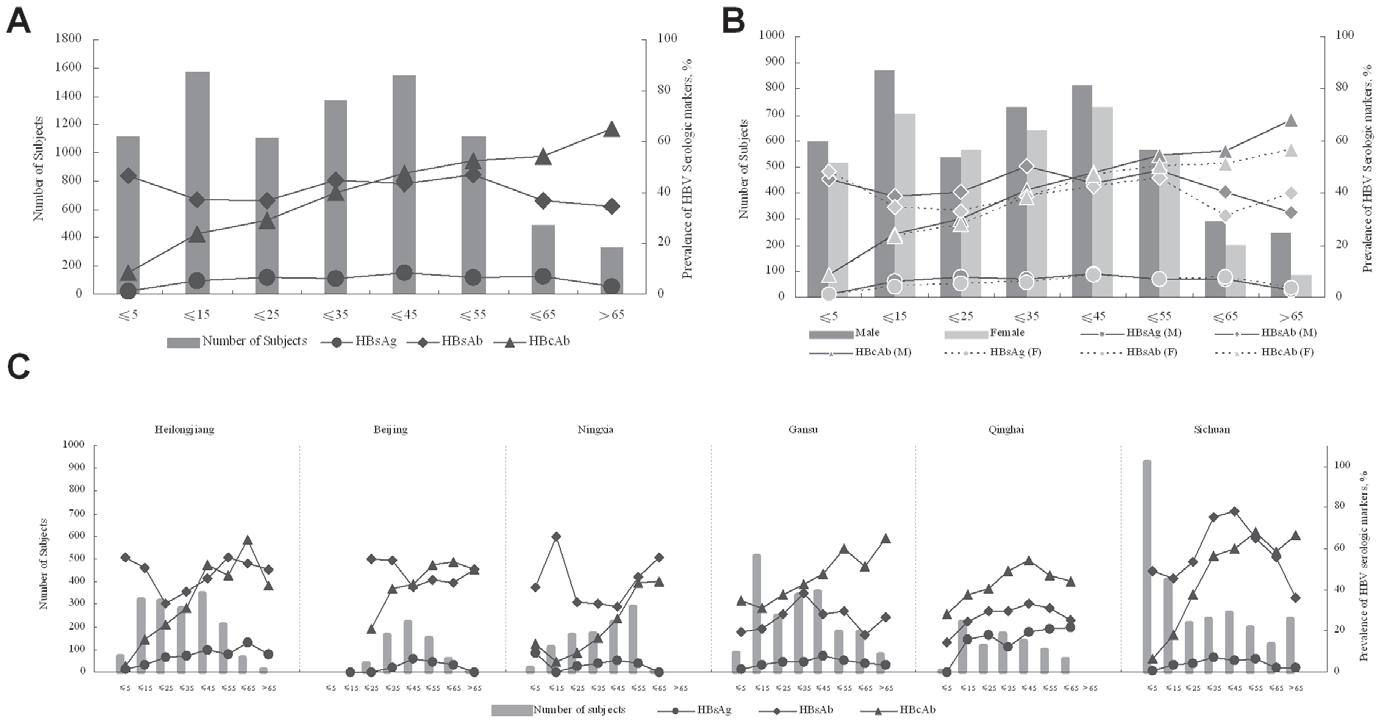
Prevalence of HBV serologic markers in China. Bars represent the number of subjects in each group indicated by left vertical axis, and the points represent the prevalence in each group indicated by right vertical axis. The horizontal axis represents the age ranges. A, Overall characterization of HBV serologic markers by age range group. B, Overall characterization of HBV serologic markers by gender, M, male, F, female. C, Regional characterization of HBV serologic markers by age group.

The overall prevalence of HBsAg was 5.84% (512 positive) in 8,762 subjects, which is significantly lower than that reported in 1992 (9.8%) [31], suggesting that the population of HBV carriers is decreasing. In the ≤5 group, the prevalence of HBsAg was 1.16%, and the prevalences of HBcAb and HBsAb among this group were 8.69% and 46.77%, respectively. These results indicate that strong efforts have been made in younger children since the HBV infant vaccination was introduced in 1992 and integrated into the national immunization program in 2002 (Fig. 2A). Moreover, HBIG and HBV vaccine treatment for newborns of HBV-infected mothers at delivery could also contribute to the significant reduction of vertical or perinatal transmission of HBV. In the >60 group, HBsAg prevalence was also low, probably due to deaths caused by HBV-related cirrhosis and hepatocellular carcinoma in this group [33] (Fig. 2A).

The prevalence of HBsAb was 41.31% overall (Fig. 2A), which was different from the prevalence figures for anti-HAV and anti-HEV (see following section). This percentage showed a relatively stable level across all age groups, which suggested that HBsAb may not be as stable as are anti-HAV and anti-HEV. Alternatively, the increase in anti-HAV and anti-HEV may be due to repeated contact with HAV and HEV. Taken together, our data raised a question as to whether the current HBV vaccination program may be inadequate. It is of note that although the HBsAg showed significantly lower prevalence in young generation in our study, HBsAb was still at 46.77% (Fig. 2A), which is very close to over all the average (41.31%). Other factors that might contribute to HBsAb prevalence, including infection acquired through vaccination, are large numbers of children delivered outside hospital settings, popular movements opposing HBV vaccination coverage, or procedural factors including the efficiency of the immunization program and the quality of the vaccine.

However, HBcAb prevalence increased with age from 8.69% in the ≤5 group to 65.16% in the >65 group, with an overall prevalence 35.92% (Fig. 2A). In contrast to HBsAb, which can be caused by HBV infection or by vaccination, HBcAb results only from HBV infection. Comparing HBcAb and HBsAb by age, in the younger groups, HBcAb prevalence was much lower than was that for HBsAb, suggesting that HBsAb was mainly caused by the HBV vaccine. In older groups, HBcAb prevalence increased and became higher than that for HBsAb, with trends for the intersecting in the age groups spanning 36–45 years of age (Fig. 2A). This indicates that both HBsAb and HBcAb were mainly caused by latent HBV infection. Furthermore, the difference between prevalence trends for HBsAb and HBcAb raises the possibility that HBsAb has declined faster than HBcAb, suggesting a need for precise evaluation of the maintenance of HBsAb derived from the HBV vaccination program and opens the possibility that re-vaccination may be required in older populations.

When the overall prevalence of HBV serologic markers were stratified by gender, the prevalence of HBsAg, HBsAb and HBcAb were 6.24%, 43.53% and 37.76% respectively in male subjects, and were 5.38%, 38.80% and 33.82% respectively in female subjects. All three serologic markers showed higher prevalence in male than that in female, although the prevalence of HBsAg showed mildly higher in female in the subjects older than 45 and the prevalence of HBsAb in female was higher in the >65 group (Fig. 2B).

HBV serologic markers showed regional characteristics. In Beijing, Ningxia, Gansu, and Sichuan, HBsAg prevalence was consistent with the overall situation mentioned above. In Heilongjiang and Qinghai, however, HBsAg prevalence was higher than in the other regions (Fig. 2C). HBsAb prevalence was higher in younger age groups in Heilongjiang and Sichuan, which may suggest a better vaccination program for children in these regions. HBcAb prevalence is high in older groups from all regions investigated. Especially in Qinghai and Gansu, HBcAb prevalence remained higher than that of HBsAb, which suggests that the HBV vaccination is insufficient in these regions and the prevalence of HBsAb together with HBcAb mainly resulted from latent HBV infection (Fig. 2C).

### Prevalence of Anti-HAV

**Figure 3:**
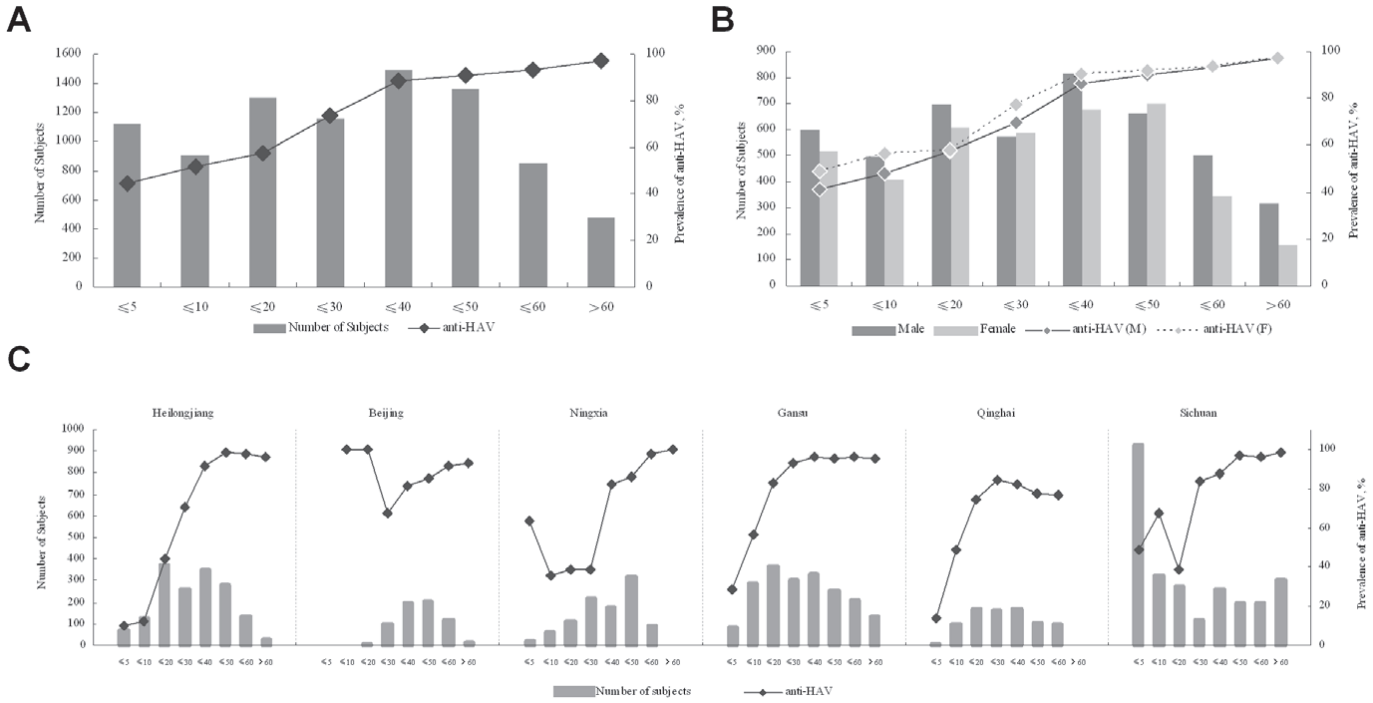
Serological features of anti-HAV prevalence in China. Bars represent the number of subjects in each group indicated by left vertical axis, and the points represent the prevalence in each group indicated by right vertical axis. The horizontal axis represents the age ranges. A, Overall characterization of anti-HAV by age group. B, Overall characterization of anti-HAV by gender, M, male, F, female. C, Regional characterization of anti-HAV by age group.

The overall prevalence of anti-HAV in 8,762 subjects was as high as 72.87%. Anti-HAV prevalence in ≤5, ≤10, ≤20, ≤30, ≤40, ≤50, ≤60, and >60 years age groups was 44.71%, 51.88%, 57.64%, 73.51%, 88.32%, 91.12%, 93.51%, and 97.27%, respectively. The prevalence of anti-HAV showed an increase from younger groups (44.71%) to older groups (97.27%) (Fig. 3A). This high anti-HAV prevalence may result from the long history, large population, inadequate sanitation, and poor hygiene of China that may favor HAV circulation and transmission [11]. The high prevalence in the ≤5 group may be partially due to the HAV vaccination. Although the prevalence of anti-HAV in male was lower than that in female, the over all prevalence of anti-HAV was very close, that was 72.17% and 73.67% respectively (Fig. 3B).

For all regions investigated, anti-HAV prevalence showed a similar tendency, increasing gradually from the ≤20 to the >60 groups. In the ≤5 and the ≤10 groups, the prevalence of anti-HAV varied regionally. In Heilongjiang, Gansu, and Qinghai levels were consistently lower. In Beijing and Ningxia, the high prevalence of anti-HAV may be due to a sampling bias. In Sichuan, the prevalence of anti-HAV was 48.60% of 930 subjects in the ≤5 group and 67.49% of 323 subjects in the ≤10 group (Fig. 3C).

### Prevalence of Anti-HEV

**Figure 4:**
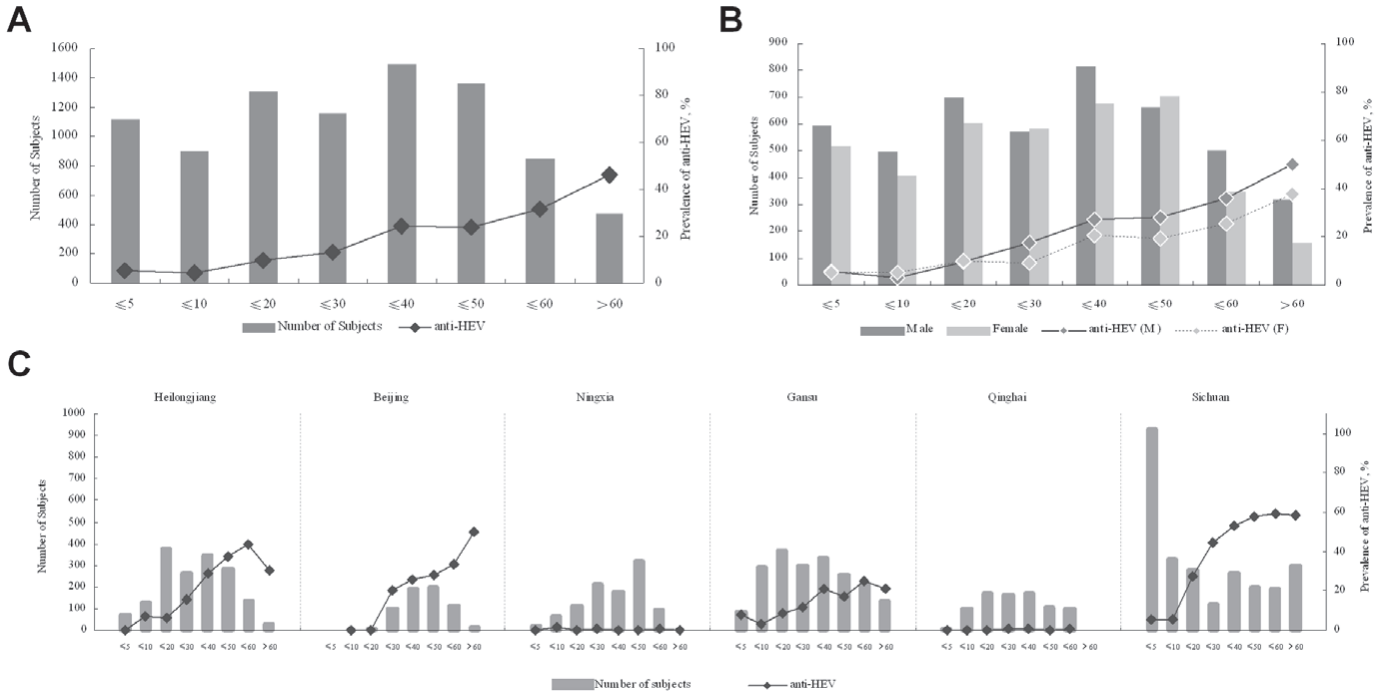
Serological features of anti-HEV prevalence in China. Bars represent the number of subjects in each group indicated by left vertical axis, and the points represent the prevalence in each group indicated by right vertical axis. The horizontal axis represents the age ranges. A, Overall characterization of anti-HEV by age group. B, Overall characterization of anti-HEV by gender, M, male, F, female. C, Regional characterization of anti-HEV by age group.

The overall prevalence of anti-HEV in 8,762 subjects was as high as 17.66%. Anti-HEV prevalence in ≤5, ≤10, ≤20, ≤30, ≤40, ≤50, ≤60, and >60 age groups was 5.38%, 4.20%, 9.75%, 13.20%, 24.23%, 23.70%, 31.52%, and 45.91%, respectively (Fig. 4A). For subjects younger than 20, the prevalence of anti-HEV in female subjects showed mildly higher than that in male, while for those subjects older than 20, the prevalence in male became higher than that in female (Fig. 4B). Similar to anti-HAV, the prevalence of anti-HEV increased significantly in the ≤10 and ≤20 age groups and then increased gradually with age in Heilongjiang, Beijing, Gansu, and Sichuan. However, the average level was much lower than was that for anti-HAV.

In Ningxia and Qinghai, the prevalence of anti-HEV was extremely low regardless of age, with an average prevalence of 0.40% and 0.37%, respectively. This evidence corresponds with our ongoing study showing that swine are a major reservoir and source of HEV in China [30]. Ningxia has a primarily Muslim population, and Qinghai is a Tibetan area; both of these populations have little contact with swine due to the nature of their spiritual practices (Fig. 4C).

## Discussion

Hepatitis is a major public health burden in China [31], yet complex epidemiological examinations of hepatitis A, B, C, D, or E have very rarely been performed. Although viral hepatitis is an “old” problem, the understanding of viral hepatitis remains vague even as attention is paid to newly emerging and highly pathogenic viral diseases that have been the recent focus of public health efforts. Given the vast number of viral hepatitis cases in China [31], we should not ignore these “old” diseases. In this study, our epidemiologic survey examined hepatitis A, B, C, and E in 8,762 individuals distributed throughout six regions in China to demonstrate the general status of viral hepatitis infection on a regional level and with age-specific profiles to benefit worldwide hepatitis surveillance.

HAV and HEV infection may result in acute hepatitis with a certain death rate [11], [34]. Our data showed these two viruses share a similar serological prevalence in China. The overall positive rates are very high in Han ethnic areas, and the rate tends to increase with age, suggesting that common transmission routes may determine common prevalence patterns for these two viruses. This evidence may contribute to mathematic models for other infectious diseases in the future [35]. With regard to hepatitis E, based on our decades-long surveillance, we recognized that HEV transmission in China is closely related to the presence of swine (BI's unpublished data). In areas where people live with swine, hepatitis E outbreaks are constant. In contrast, in areas in China where people do not live with swine, hepatitis E rarely occurs, which suggests that swine constitute a major reservoir and source of HEV. Although many studies have reported the isolation of HEV from swine and other animals [36]–[38], our data are the first to demonstrate that the prevalence of HEV is related to culture and religion. However, for hepatitis A, our data show no differences in prevalence related to ethnicity. Although our study subjects included a certain number of HAV vaccinated young individuals, our data showed that among people older than 20 years, the anti-HAV prevalence is more than 60%, and this rises to nearly 100% in the group over 60 years. These data suggest that the anti-HAV response detected in a large number of Chinese people reflects a latent infection.

Large-scale studies on the prevalence of HCV are not well documented in many countries, especially in developing countries. Worldwide, 3% of the population has been estimated to be infected by HCV [22]. In China, specialists and clinicians doubt this rate, because both epidemiologic studies and random clinical cases never fit this rate. Although this study had some deficiencies in subject sampling, our data showed that the overall average prevalence of anti-HCV was 0.58% in China. Together with data from our ongoing nationwide HCV serological survey (in which the overall anti-HCV prevalence is less than 0.5% among more than 80,000 Chinese subjects (BI's unpublished data), these findings lead us to believe that the exact HCV prevalence is much lower than the WHO estimation indicates. HCV was identified in 1989, and HCV screening in blood began in the 1990s, a practice that may significantly reduce the transmission rate of HCV by blood transfusion and other sources of iatrogenic infection. Therefore, it is reasonable that the transmission and spread of HCV can be controlled and prevented by strict blood screening and other procedural measures.

A total of 9.8% of the Chinese population were estimated to be carriers of HBV in the 1992 national wide HBV survey [31]. Sixteen years have passed since that survey. So what are the current features of HBV prevalence in China? HBV infant immunization was introduced in 1992 and integrated into the national immunization program in 2002. What is the effect of HBV vaccine and what is the actual status of the HBV immunization program? Our report answered these questions as well. Generally, the overall HBsAg prevalence was 5.88% in this study, which is very close to the nationwide HBV survey performed on more than 80,000 subjects in 2006 [21]. In the ≤5 group, the prevalence rates of HBsAb, HBsAg, and HBcAb were 46.77%, 1.16%, and 8.69%, respectively, which suggests that the antibody level of most individuals is raised by HBV vaccination. With regard to the question of why only 46.7% individuals in this group are positive for HBsAb, the national immunization program has not really responded to this question well. Quality problems with the HBV vaccine and lack of response to the HBV vaccine are likely the main reasons. The average HBsAb prevalence is 41.90%, which is different from prevalences for anti-HAV and anti-HEV. The data showed a tendency for HBsAb to decline with increasing age, suggesting that HBsAb may be easily degraded. Our data also raise a query as to whether the three doses of HBV vaccine given in childhood are enough to maintain HBV prevention throughout life. Overall, in the present study, HBcAb tended to increase with increasing age. Together with the pattern of HBsAg prevalence, these data suggest that for a majority of Chinese adults, HBsAb resulted mainly from latent infection.

This was a cross-sectional study performed on large-scale population. The data-collection process had some shortcomings, including the failure to collect vaccination histories for subjects and the problem of bias in sampling. Nevertheless, this study provided much important information concerning the prevalence of hepatitis A, B, C, and E in Sichuan, Qinghai, Ningxia, Gansu, and Helongjiang Provinces as well as Beijing city of China, and we believe our data reflect a close approximation to the national wide exact situation of hepatitis infections.

## Materials and Methods

### Ethics Issues

All aspects of the study were performed in accordance with the national ethics regulations and approved by ethics committee of CDC, China as well as local CDC in related province. Study participants were informed of the study's purpose and of their right to keep information confidential. Written consents were obtained from the participants involving in this study and approved by ethics committee of local CDC.

### Study Subjects and Specimen Collection

**Figure 5:**
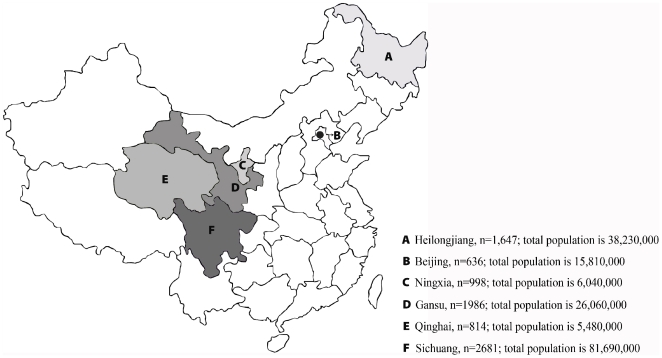
Location of the six study areas in China. “n” represents the number of subjects in each area. Statistical data on the population of each area are from National Bureau of Statistics of China, updated in 2007 when samples were collected.

The target subjects in this study were the normal Chinese individuals which were selected to be as representative of the demographic characteristics as possible in regarding areas of China. Considering the prevalence of related serologic makers of hepatitis A, B, C, and E, a total of 8,762 blood samples were obtained randomly from physical examination participants distributed throughout Sichuan, Qinghai, Ningxia, Gansu, and Helongjiang Provinces as well as Beijing city in 2007, in which the number of male subjects was 4661 and the number of female subjects was 4101. As multiple ethnicities live in China, and hepatitis prevalence is different among regions, sampling regions included two minority areas (Ningxia and Gansu). The vaccination history of subjects was not considered. A total of 1,647 samples were collected from Heilongjiang, 636 from Beijing, 998 from Ningxia (a Muslim area), 1986 from Gansu, 814 from Qinghai (a Tibetan area), and 2,681 from Sichuan (Fig. 5). Serum was separated at local laboratories, transported to the National Hepatitis Laboratory at the Chinese Centers for Disease Control (CDC) in Beijing, and stored at −40°C until laboratory testing was performed.

### Age Grouping of Subjects

All subjects from each region were grouped by age range: ≤5, ≤10, ≤20, ≤30, ≤40, ≤ 50, ≤60 and >60 years of age for epidemic analysis (“≤” indicated beginning with the older than the end point of last age range). For HBV epidemiology, age grouping was based on the national immunization policy for HBV that was first introduced in 1992 and integrated into the national immunization program in 2002. Thus, subjects were grouped by age ranges of ≤5, ≤15, ≤25, ≤35, ≤45, ≤55, and >65years of age.

Due to random sampling, the number of subjects in each age group was variable. In particular, Beijing had no subject in the ≤5 group, and only one and six subjects in the ≤10 and ≤20 groups, respectively. In Ningxia, the >60 group had one subject. In Qinghai, seven subjects were included in the ≤5 group, and the >60 group had no subjects. In Sichuan 930 subjects were in the ≤5 group, which was significantly more than the number in the same age groups in other regions. With regard to the age groupings for HBV epidemiology, the distribution of subjects in the ≤5 group was the same as that mentioned above. Deficiencies in other age groups included only one subject in the ≤15 group and four in the >65 group. In Ningxia and Qinghai, no subjects were included in the >65 group.

### Laboratory Testing

All serum specimens were tested in the National Hepatitis Laboratory, Institute for Viral Disease Control and Prevention at the China CDC. A detailed laboratory testing protocol was used, including a retest of specimens with inconsistent results. Testing kits were selected based on an evaluation of available enzyme-linked immunosorbent assay (ELISA) kits in China and comparison with Abbott enzyme immunoassay reagents (Illinois, USA) using a panel of 200 reference serum specimens. ELISA diagnostic kits for HBV serologic markers, anti-HAV, anti-HCV, and anti-HEV IgG were all purchased from Beijing Wantai Biological Pharmacy Enterprise Co., Ltd. (Beijing, China) and used according to the manufacturer's instructions.

### Data Analysis

All serological prevalence indices and figures in this study were calculated by Microsoft Office Excel 2003.
